# Prediction of subthalamic stimulation efficacy on isolated dystonia via support vector regression

**DOI:** 10.1016/j.heliyon.2024.e31475

**Published:** 2024-05-17

**Authors:** Yunhao Wu, Yan Li, Hongxia Li, Tao Wang, Peng Huang, Yiwen Wu, Bomin Sun, Yixin Pan, Dianyou Li

**Affiliations:** a Department of Neurosurgery, Center for Functional Neurosurgery, Ruijin Hospital, Shanghai Jiao Tong University School of Medicine, Shanghai, China; b Department of Radiology, Ruijin Hospital, Shanghai Jiao Tong University School of Medicine, Shanghai, China; c Department of Neurology and Institute of Neurology, Ruijin Hospital, Shanghai Jiao Tong University School of Medicine, Shanghai, China

**Keywords:** Isolated dystonia, Subthalamic nucleus, Deep brain stimulation, Support vector regression

## Abstract

**Introduction:**

Deep brain stimulation (DBS) of subthalamic nucleus (STN) has been well-established and increasingly applied in patients with isolated dystonia. Nevertheless, the surgical efficacy varies among patients. This study aims to explore the factors affecting clinical outcomes of STN-DBS on isolated dystonia and establish a well-performed prediction model.

**Methods:**

In this prospective study, thirty-two dystonia patients were recruited and received bilateral STN-DBS at our center. Their baseline characteristics and up to one-year follow-up outcomes were assessed. Implanted electrodes of each subject were reconstructed with their contact coordinates and activated volumes calculated. We explored correlations between distinct clinical characteristics and surgical efficacy. Those features were then trained for the model in outcome prediction via support vector regression (SVR) algorithm and testified through cross-validation.

**Results:**

Patients demonstrated an average clinical improvement of 56 ± 25 % after STN-DBS, significantly affected by distinct symptom forms and activated volumes. The optimal targets and activated volumes were concentratedly located at the dorsal posterior region to STN. Most patients had a rapid response to STN-DBS, and their motor score improvement within one week was highly associated with long-term outcomes. The trained SVR model, contributed by distinct weights of features, could reach a maximum prediction accuracy with mean errors of 11 ± 7 %.

**Conclusion:**

STN-DBS demonstrated significant and rapid therapeutic effects in patients with isolated dystonia, by possibly affecting the pallidofugal fibers. Early improvement highly indicates the ultimate outcomes. SVR proves valid in outcome prediction. Patients with predominant phasic and generalized symptoms, shorter disease duration, and younger onset age may be more favorable to STN-DBS in the long run.

## 1 Introduction

The history of subthalamic nucleus (STN) deep brain stimulation (DBS) can be traced back to the success in treating patients with Parkinson's Disease (PD) in the 1990s [1]. Expanded application of STN-DBS has also been proven effective in refractory isolated dystonia over the recent decades [2]. Ostrem et al. observed over 70 % average motor symptoms improvement in 20 patients with isolated dystonia after 36 months of STN chronic stimulation [3]. Such safety and persistency of therapeutic effect in primary dystonia had also been demonstrated in a ten-year follow-up study [4]. Since findings from previous studies and reports indicated noninferior or even superior efficacy of STN-DBS compared to globus pallidus interna (GPi) DBS, this treatment could probably serve as a remedy for patients who have failed traditional surgical procedures [[5], [6], [7]].

DBS candidates with high therapeutic value need to be appropriately evaluated and identified. However, evidence in the current literature lacks sufficient indications for potential surgical benefits in dystonia patients. Efficacy differs greatly among individuals, and nearly a quarter of patients still fail to respond effectively to DBS (empirically defined as less than a 25 % decrease in BFMDRS scores postoperatively) [2,8]. Such disparity in therapeutic effects calls for the demand to probe the underlying factors, including disease characteristics, electrodes’ coordinates, applied stimulating parameters, etc.

In this study, we enrolled a cohort of patients diagnosed with isolated segmental/generalized dystonia and received bilateral STN stimulation at our center. Regular follow-up assessments lasted over one year. Through analyses of various clinical characteristics, we intended to explore the main factors that were associated with STN-DBS outcomes. Further, a prediction model was established via a machine learning method, support vector regression (SVR) algorithm, to train the above features. Results from this conducted study would potentially offer us a better understanding of STN-DBS on isolated dystonia and more practical guidance on patient screening and treatment options in the future.

## 2 Methods

### 2.1 Participant inclusion

We performed a prospective study including consecutive patients diagnosed with idiopathic isolated dystonia and underwent STN-DBS between 2018 and 2021 in our clinical center. This study design was approved by the ethics review board and written informed consent was obtained from all individual participants. Patients met the following inclusion criteria were eligible: definite diagnosis of isolated segmental/generalized dystonia by experienced neurologists, according to the guideline of the 2013 Movement Disorder Society Consensus [9]; ages between 18 and 75; and unsatisfactory alleviation to adequate oral medication or botulinum toxin injection. Exclusion criteria contained patients with other neurological or psychiatric comorbidities; brain surgery histories; abnormal structural findings (e.g., brain atrophy, detected by MRI); severe cognition impairment (Mini-mental State Examination scores <24); and those who failed to complete the planned follow-up visits.

A total of 32 patients (16 females and 16 males) with a median age of 49.5 years (25th percentile: 28.0, 75th percentile: 61.5) (Shapiro-Wilk test W = 0.915, p = 0.016) were enrolled in our study. Burke–Fahn–Marsden Dystonia Rating scale (BFMDRS) was applied for quantitative assessment of dystonia severity. The scale is composed of two sections, Movement and Disability Scale, which are sums of severity scores of nine body regions and degrees of daily function impairment respectively [10]. Disease severity was evaluated and scaled through videos by another physician independently. All videos were randomly evaluated by the physician, so their visiting time points were unnoticed. We divided the patients into different subgroups according to their distinct clinical manifestations and surgical outcomes. Patients with predominant phasic symptoms were differentiated from tonic ones. These two types are differentiated by involuntary dystonic muscle activities. Tonic dystonia is characterized by sustained abnormal postures, while phasic dystonia, is featured by hyperkinetic rhythmic activity or irregular brief jerking motion. Patients with mixed manner would be further evaluated which component is dominant or which component presented in wider body regions. Additionally, based on varied body distributions, they were categorized as segmental dystonia (characterized by blepharospasm and other cranial muscles affected, which were also described as Meige's syndrome [11]), and generalized dystonia (trunk and extremities affected). (Detailed information was demonstrated in the Supplementary table).

### 2.2 Surgical procedures, programming, and evaluations

The surgical procedures of bilateral STN-DBS have been described before [12]. In brief, we obtained preoperative 3T MRI scans where the target structures could be demonstrated. After the stereotactic frames were attached to the heads, frame-based CT scans were performed and coregistrated to preoperative images via surgical planning software (Leksell SurgiPlan), and then coordinates and trajectory angles of the target were confirmed. DBS procedures were performed under general anesthesia for their merits of safety and accuracy in electrode position. Quadripolar microelectrodes (model 3387, Medtronic Minneapolis, MN, USA/1210–40, SceneRay, Suzhou, China/L302, PINS, Beijing, China) were inserted into the dorsolateral area of STN, being connected to the neurostimulator placed in the subclavicular region subsequently. The surgery was performed either in one procedure or in two stages, which were optional for patients. In this study, the proportion is 26/6 (one vs two-stage procedure). In staged operations, electrodes were first inserted, connected with temporal external neurostimulators. Permanent pulse generators (IPG) were implanted within a couple of days after confirming efficacy from trial stimulation. Neurostimulators were activated right after the electrodes’ implantation, initially using bipolar configurations and low amplitude (130 Hz, 60μs–90 μs, and 1.5 V–2.0 V) for adaptation. Within one week, patients underwent the first programming by an experienced neurologist, to make sure the parameters were adjusted to reach the maximal clinical outcome while avoiding acute adverse effects. Postoperative assessment was uniformly performed at three time points at least: one week, one month, and over one year of continuous STN stimulation. The final assessment should satisfy the condition of stable symptom relief in the last three months. Additional assessment and programming could be scheduled if necessary or patients required. Any surgical complications, device, and stimulation-related neurological sequelae would be recorded as adverse events.

### 2.3 Localization of DBS electrodes

The electrode leads were reconstructed and visualized using Lead-DBS v2.5 software (https://www.lead-dbs.org) [13]. All postoperative CT scans were registrated to the preoperative T1-weighted images and normalized to standard Montreal Neurological Institute (MNI) space (ICBM 2009b NLIN) with Advanced Normalization Tools (ANTs) [14]. After the quality check of the coregistration, Brainshift Correction module was applied if needed for reducing substantial brain shift or pneumocephalus generated by the operations. Electrodes trajectories were reconstructed by PaCER method and the contact positions were further refined manually [15]. The localizations of the electrodes were finally visualized in the three-dimensional template space, with DISTAL Minimal and STN-Subdivisions atlases added displaying the relative position of electrodes to the nucleus [16,17]. For all subjects, Lead group toolbox was applied for group analysis including calculating MNI coordinates of both-side activated (cathodic) contacts and measuring their Euclidean distances to the surface of STN motor territory. Particularly, when there was more than one activated contact on a single electrode, mean values were calculated.

### 2.4 Construction and validation of VTA-based outcome map

For each subject, the volumes of tissue activated (VTAs) were simulated given parameters (voltage) of activated contacts and normalized to MNI space using the previous transform field. After reconstructing all electrodes and VTAs in standard space, voxels within a subject's VTAs were labeled values of the surgical outcome (BFMDRS improvement), thus transforming binary VTAs into weighted ones (right VTAs were flipped to the left side). An outcome heat map was constructed by fusing all individual weighted VTAs, where each voxel in the map would be labeled with arithmetic mean value of covered VTAs. We presumed that voxels corresponded to more covered VTAs with higher average clinical outcomes would result in a better location for stimulation. A prediction value from the outcome map was computed as the mean value of labeled voxels covered by the individual VTA. To evaluate the reliability of the generated VTA outcome map, the individual predicted values were both verified in the original, 32-sample-based training set and a new 10-subject test set. The test set was selected independently from the original training cohort (Supplementary Table 1b). The ten new dystonia patients also met the same inclusion criteria as others, receiving over one-year postoperative visits. The predicted value from a new individual VTA would be defined as its mean value of voxels within the constructed map volume. We calculated the Pearson correlation coefficient from the correlation between predicted and actual measured values, of which p < 0.05 was considered statistically significant.

### 2.5 VTA-based deterministic fiber tracking

Tractography was applied in Lead-DBS to explore the probable white fiber in suppressing dystonic movements. Here, voxels within the fused VTA map were extracted as the seed for deterministic fiber tracking, and calculation was implemented on a diffusion-weighted dataset of ‘Structural group connectome 32 Adult Diffusion HCP subjects GQI’ [18].

### 2.6 Construction of outcome prediction model

SVM is a supervised learning algorithm to recognize patterns, aimed at finding a max-margin separator hyperplane for classification. SVR is extended from SVM for continuous variables prediction, based on the same principle except for a few differences (to find a minimum interval hyperplane containing maximum samples while balancing the prediction error) [19]. As one of the classic machine learning methods, SVR possesses the advantages of a high level of precision, generalizability, and excellent performance in handling highly non-linear data using kernel trick. Here, an outcome prediction model was constructed via SVR based on different clinical characteristics. Before calculation, all variables were normalized to a section ranging from 0 to 1 (dichotomous data was defined as 0 or 1), from which the transformation was later applied to the predicted values. Model training and evaluating were implemented by the MATLAB 2020a-based LIBSVM toolkit [20], using embedded svmtrain (linear kernel for this projection) and svmpredict functions. Features for model training included preoperative demographic characteristics, prediction values from the VTA-based outcome map, and one-week motor improvement postoperatively. The output label was defined as the long-term clinical outcome (percentage of overall BFMDRS decrease) in the latest assessment. Leave-one-out cross-validation was applied to identify the optimal regularization parameter C (penalty coefficient) and evaluate the ultimate model performance. Specifically, in each loop, all samples were assigned to a training set (31/32) and left one (1/32) as a test sample for model learning and evaluation. Training and test sets were reassigned in the next loop, repeating 32 times. Learning performance was finally evaluated by calculating mean absolute error (MAE), mean square error (MSE), and correlation coefficient (r) between measured and predicted values from 32 times of leave-one-out cross-validation. By ranking the components of the weight vectors, we would be able to evaluate distinct features contributing to the decision value of the model. In the next step, we tested the outcomes from several different models to find out which features were best fitted for model estimation. In these models, different numbers of features, from the highest-weighted to the lowest-weighted ones, were successively applied for estimation. Optimal features would be picked from the SVR model which presented the lowest MSE value.

### 2.7 Statistical analysis

For continuous variables, we used Shapiro-Wilk test to examine their statistical distributions. Independent two-sample *t*-test was applied for group comparisons if variables were normally distributed, elsewise Mann-Whitney *U* test was applied. Pearson correlation analysis was applied to investigate the relationship strength between therapeutic efficacy and clinical characteristics. P values less than 0.05 (two-tailed) were judged to be significant.

The significance of the assessment indicators for SVR performance was demonstrated using a permutation test (permutation times n = 1,000, significant level α = 0.05). In each test before training, the data labels were randomly permuted and such procedure was repeated 1000 times to determine whether the performance occurred by chance (probability of more than 5 %).

## 3 Results

### 3.1 Surgical outcomes and stimulation parameters

Patients presented an overall BFMDRS score reduction of 56 ± 25 % (Shapiro-Wilk test W = 0.944, p = 0.096) in the latest follow-up visit after STN-DBS (the shortest follow-up time: 12 months; the longest follow-up time: 24 months). 44 % (14 of 32) of them had over 70 % symptom improvement in the long term, which was classified as superior outcome group, and the rest proportion of moderate outcome group (25%–69.9 % improvement) was 41 % (13 of 32) and 16 % (5 of 32) in inferior outcome group (less than 25 % improvement) (Supplementary Table 1 and 2).

The three groups did not differ in the optimal stimulation parameters. Their average amplitude, width and frequency are 2.93 ± 0.62 V (p = 0.131), 67.19 ± 15.71 μs (p = 0.297), and 141.56 ± 15.32 Hz (p = 0.500) in the right electrodes, and 2.94 ± 0.70 V (p = 0.335), 66.25 ± 16.21 μs (p = 0.545), and 142.66 ± 16.90 Hz (p = 0.539) in the left electrodes (Supplementary Table 2 for stimulation configurations and parameters in detail).

### 3.2 Adverse events

No serious adverse events occurred during the whole study process. Stimulation-related adverse events were reported in four patients. In generalized dystonia, one reported increased gait instability, and dysphagia occurred in another patient. The other two with Meige syndrome reported slight dysarthria, which was transient and could be alleviated by parameter adjustment. However, the avoidance of the slurred speech also diminished the maximum improvement of blepharospasm symptoms.

### 3.3 Association between surgical outcomes and clinical features

**Fig. 1:**
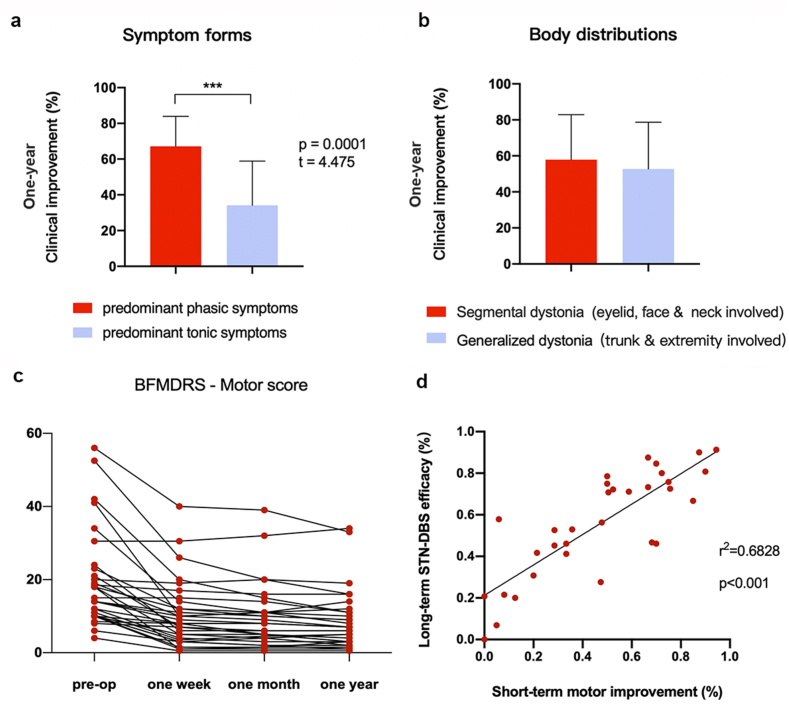
STN-DBS outcomes in distinct subtypes of isolated dystonia and their alterations during long-term observation. STN-DBS outcomes (≥one-year clinical improvement) comparisons in distinct symptom forms (a) and body distributions (b) were presented using an independent *t*-test. Evaluations of all patients' BFMDRS motor scores were demonstrated before and after STN-DBS (c). A significant correlation was found between patients' short-term (one week) and long-term (over one year) clinical improvement (d).

In groups of distinct symptom forms, patients with predominant phasic dystonia presented significantly higher efficacy than patients with predominant tonic dystonia (67 ± 17 % vs 33 ± 26 %; Levene test F = 2.113, p = 0.156; two sample *t*-test p＜0.001). There were no significant differences in the outcome between patients with distinct body distributions (eyelid, face & neck affected vs trunk & extremities affected, Levene test F = 0.160, p = 0.692; two sample *t*-test p > 0.05), demonstrated in Fig. 1a&b.

In terms of other continuous clinical variables, including age of onset, disease duration, and baseline BFMDRS scores, etc., none of them were found significantly correlated to the ultimate clinical outcomes. However, we found that most patients demonstrated considerable motor symptom reduction shortly after STN-DBS (Fig. 1c). By analyzing the altering trends of surgical efficacy in different phases, a significant correlation was found between the long-term surgical outcome (BFMDRS total scores decrease in the latest visit/baseline BFMDRS total scores) and short-term motor improvement (postoperative BFMDRS movement scale scores decrease in one-week/baseline BFMDRS movement scale scores) (Pearson r^2^ = 0.6828, p＜0.001, Fig. 1d).

### 3.4 Association between surgical outcomes and electrodes’ parameters

**Fig. 2:**
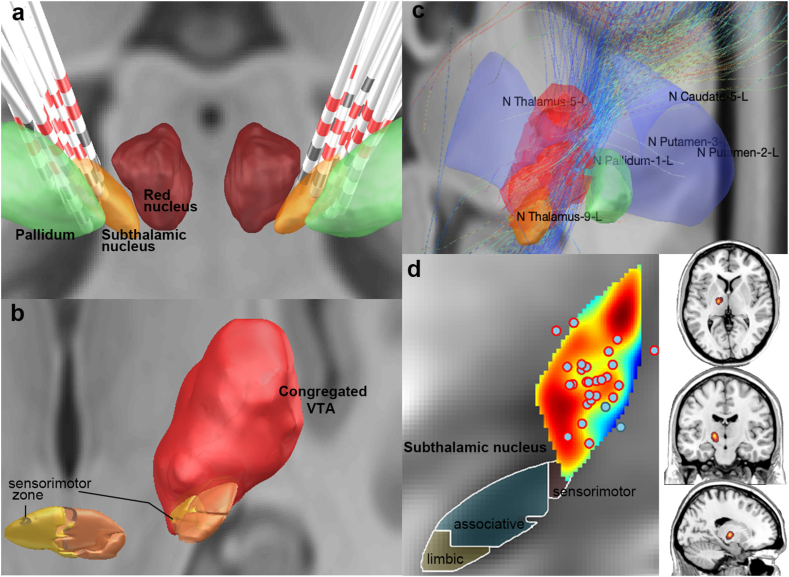
Reconstruction of electrodes and volumes of tissue activated. Electrodes of all subjects were reconstructed through Lead-DBS (a) (https://www.lead-dbs.org). DISTAL Minimal and STN-Subdivisions atlases were displayed on the template space. Activated contacts were highlighted in red (a&d). Congregated volumes of tissue activated (VTA) (b) and deterministic fiber tracking based on seeds of VTA (c) were demonstrated (right VTAs were flipped to the left side). VTA-based outcome heatmap was displayed on a 2D plot (coronal view) and overlaid on T1-weighted imaging (d).

64 electrodes in 32 subjects were projected to MNI standard space, where the individual coordinates of the active contacts relative to the midcommissural point of the AC-PC line were defined: 15.20 ± 1.08 mm lateral, 11.75 ± 1.71 mm posterior and 9.13 ± 2.49 mm inferior. On the two-/three-dimensional electrode reconstruction images, their activated contacts and VTAs were mainly located at the dorsal posterior region of STN (Fig. 2a&b). Within the VTAs, parts of the fibers were received from the basal ganglia, traveled dorsal through STN, and projected to the thalamus demonstrated by the results from deterministic fiber tracking (Fig. 2c).

X and Y coordinates of activated contacts did not differ among groups with different surgical outcomes (P_X_ = 0.184, P_Y_ = 0.390), however, Z coordinates were larger in the superior outcome group than the other two groups (P_Z_ = 0.040), indicating a trend of more dorsal activated volume in reaching optimal outcome. Additionally, the coordinates of the activated contact did not differ in segmental and generalized dystonia groups (P_X_ = 0.359, P_Y_ = 0.505, P_Z_ = 0.165). Moreover, distances between activated contacts and the surface of STN motor territory were calculated, demonstrating no significant correlations with the surgical outcomes (Pearson p = 0.0764).

**Fig. 3:**
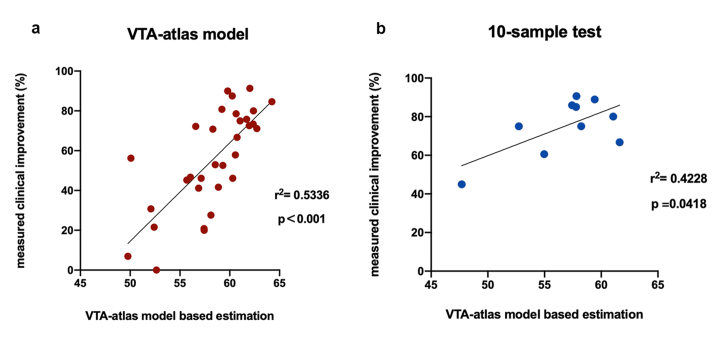
Validation of VTA-based prediction model in primary and new samples. Pearson correlation analyses demonstrated significant correlations between VTA-atlas model-based estimation and measured clinical improvement, both in the primary training cohort (p < 0.001) (a) and ten new samples (p = 0.048) (b).

In the test of the reliability of the VTA-based prediction model (Fig. 2d), values predicted from the model demonstrated a significant correlation with the authentic measured values (BFMDRS improvement), both in the initial training set (Pearson p < 0.001, r^2^ = 0.5336) and ten new subjects (Pearson p = 0.0418, r^2^ = 0.4228) (Fig. 3).

### 3.5 Performance of SVR model and feature weights

**Fig. 4:**
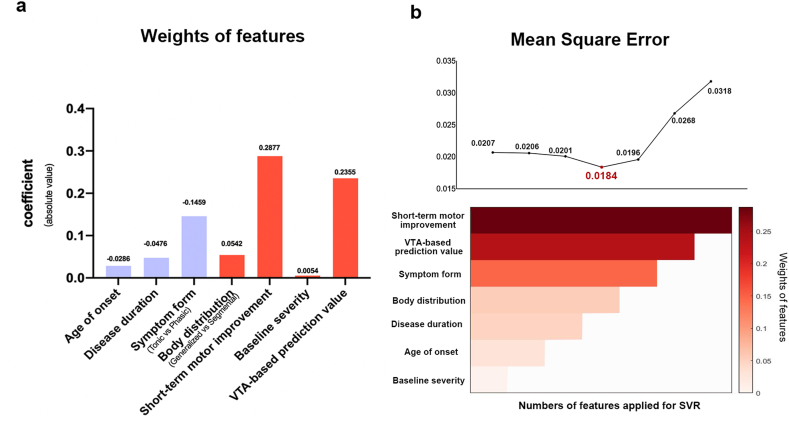
Weights of features and mean square error in distinct support vector regression models. The weights (coefficient values) of features in the support vector regression model were demonstrated in the left histogram (Positive values in red and negative values in violet) (a). Alternation of mean square error (MSE) in distinct support vector regression models with different numbers of features applied. MSE reached its lowest when features of ‘short-term motor improvement’, ‘VTA-based prediction value’, ‘symptom form’, and ‘body distribution’ were applied (b).

The leave-one-out cross-validation determined the optimal penalty coefficient C was 512, which was applied in the SVR function. Trained by seven features, SVR model achieved the accuracy of MSE = 0.0207 and the mean error of 12 ± 8 %. By ranking weighted vectors, ‘short-term motor improvement’ presented to be the heaviest weighted feature contributed to the model, with its absolute coefficient value of 0.2877, followed by the features of ‘VTA-based model’, ‘symptom form’, and ‘body distribution’ etc (Fig. 4a). Further exploration demonstrated that applying the above four features for model estimation could reach a minimum MSE of 0.0184 and a mean error of 11 ± 7 % (Fig. 4b). The clinical significance of interpreting the results presumes that if a patient is predicted to have a 25 % improvement after STN-DBS, the actual improvement rate will be largely located between 14 % and 36 % according to the SVR model.

## 4 Discussion

STN-DBS has emerged as an optional treatment strategy for isolated dystonia following GPi-DBS, while several issues remain unsolved at present. With the demand for better knowledge of the disease and improving therapeutic effects, factors need to be explored in patients who have failed to obtain adequate symptom improvement. The construction of a valid prediction model was another purpose of our study for guidance in patient screening and treatment selection in the future.

The average symptom improvement after STN-DBS in our study was 56 ± 25 % with over one-year follow-up and approximated to the efficacy of GPi-DBS (50 %–70 % previously). Consistent with the results of the previous study [5], the outcomes did not differ in patients with segmental and generalized dystonia in subgroup analysis, indicating the effectiveness of STN-DBS in distinct affected body regions. However, significantly superior outcomes were found in patients with predominant phasic dystonia than in tonic forms particularly. Phasic symptoms usually improve more rapidly than tonic forms by GPi-DBS [21], implying distinct pathophysiological mechanisms, such as pallidal oscillatory patterns [22] in the two forms. Moreover, part of early-age phasic symptoms would progress into muscle contracture in years, resulting in unsatisfactory benefits from neuromodulation and demands for adjuvant therapies. The evidence implies the dynamic pathophysiological changes in dystonia progression and its increasing difficulty to be corrected through neuromodulation. Therefore, patients with predominant phasic dystonia and shorter disease duration would be more likely to be favorable to STN-DBS.

Mechanism of chronic STN-DBS controlling dystonic symptoms would possibly refer mainly to the direct modulation of the adjacent fiber tracks instead of intranuclear neuronal activity because first, the activated volumes were found concentratedly located dorsal to the posterior region of STN after we reconstructed the electrodes, and second, no significant correlation of clinical benefit was found with distances between activated contacts to STN's motor territory. In other words, a greater stimulating impact on STN neurons didn't necessarily result in better outcomes. Also, the VTA-based outcome map constructed in our study has been tested being a potential way for outcome prediction and determining the optimal stimulating location. Pallidofugal systems may serve as the main contributing pathways for dystonic symptom suppression with electrodes analyzed in GPi-DBS [23,24]. Accordingly, VTA seeds-based deterministic fiber tracking presented part of the white fibers were anatomically corresponded to FL (the fiber traversed the internal capsule, traveled dorsal to the STN, and ended in the ventral part of the thalamus), which also implied the therapeutic effect of pallidofugal systems by STN-DBS. In this case, the ‘sweet spot’ of STN and GPi-DBS might share the common pathways [8].

The efficacy of STN in isolated dystonia has been compared to GPi-DBS in several studies, but it is still inconclusive. Some observed more favorable outcomes of STN-DBS [7], while others reported no differences [6], which was probably due to the disease heterogeneity and limited cohort sizes and categories. Nevertheless, STN-DBS still possesses its unique advantages:

First, the average stimulating intensity for symptom control in STN-DBS is lower, leading to fewer side effects and longer IPG battery life. Lower parameters might be attributed to the comparatively smaller volume of the target nucleus and stronger output from the basal ganglia to the cortex promoted by STN or the neighbored white matter [25]. Meanwhile, adverse events that had been reported in chronic GPi-DBS of dystonia including bradykinesia, dysphagia, and dysarthria [[26], [27], [28]]occurred relatively less in our patients (4/32), which could also be ameliorated by parameter adjustment. Since the side effect reductions were also accompanied by sufficient symptom improvement in patients, we supposed the distinct corresponding body regions might be closely adjacent or even overlapped. More centralized defined targeting territory and application of advanced electrodes (e.g., directional electrodes) are further necessities in acquiring more optimized benefits.

Second, most patients presented significant improvement in motor symptoms within several days of STN stimulation, while it usually took months after GPi stimulation [[29], [30], [31]]. Moreover, the degree of motor improvement in one-week follow-up was highly correlated to the long-term surgical outcomes and contributed most to the prediction values in the SVR model, which indicated dystonia responds efficiently to immediate neuromodulation effects when targeting STN.

The results could potentially affect the surgical procedure selection by patients and surgeons. In DBS surgical protocols, electrodes and IPGs could be implanted in one procedure, or two steps, leads externalization for trial stimulation and subsequent permanent IPGs implantation [32]. Given the informative results from the SVR model, staged procedure including trial stimulation would be recommended in dystonia patients with high uncertainty of the surgical benefits. In fact, in recent years, around 15 to 20 percent of dystonia patients in our center choose trial stimulation before IPGs implantation, mostly because of their concern about the little benefit from DBS with the high expense of implants. On the other hand, there are pros and cons to both options. One-procedure protocol is time-saving but risks low benefit, and the latter one can acquire more accurate assessment through trial stimulation and allows adjustment of lead position during the second procedure, though more laborious.

Trained by the most weighted features of ‘short-term motor improvement’, ‘VTA-based model’, ‘symptom form’, and ‘body distribution’, our outcome-prediction SVR model reached a maximum accuracy of 11 % in mean absolute error. Apart from features of ‘short-term motor improvement’ and ‘VTA-based model’, of which significance had been discussed before, we could infer from the model that preoperative characteristics of ‘symptom form’ and ‘body distribution’ acted as more important roles in determining the output value than features of ‘disease duration’, ‘age of onset’ and ‘baseline severity’. Acknowledged from the positive or negative coefficient value of each component in Fig. 4a, patients with phasic forms, generalized symptoms, shorter disease duration, and younger age of onset would have trends of acquiring more benefits from STN-DBS, even though some features didn't present significant correlation to the outcomes. This finding would be also valuable for patient screening before surgery, especially when a one-week assessment is not applicable.

## 5 Limitation

The first limitation in our study is feature selection. The features for model estimation were simple, and we only included clinical characteristics. Though a simpler model is more applicable across clinical centers, there is still room for accuracy improvement. Since the individual cortical atrophy patterns might influence DBS outcomes according to Gabriel et al. [33], imaging or electrophysiological characteristics (e.g., structural, or functional MRI, electroencephalograph data) need to be further explored for machine learning training. Meanwhile, a much larger cohort of patients is necessary during training to avoid model over-fitting.

Other limitations are related to the technique approach and algorithm. For instance, the tractography approaches we applied in Lead-DBS was deterministic fiber tracking using an HCP connectome template. Identifying more specific fibers requires more accurate, time-consuming, and leading-edge algorithms including probabilistic and individual tracking. Additionally, the type of electrodes commonly used in dystonia patients were constructed of each 1.5 mm-length contact (e.g., Medtronic 3387), which would not be fit for precise and refined stimulation. With more definite subregions found optimal for dystonia, advanced electrodes such as directional electrodes with shorter contact lengths, and inter-contact distances, would be preferred in future clinical practice.

## 6 Conclusion

STN-DBS is an effective procedure for isolated dystonia, with its optimal therapeutic effect probably being achieved by affecting pallidofugal pathways instead of STN motor neurons. Among various disease characteristics, patients with predominant phasic forms demonstrated superior outcomes than tonic forms. STN-DBS possesses its merits such as lower demanded parameters and shorter response time, for the early motor improvement is especially suggestive of the long-term outcomes. Therefore, STN trial stimulation before permanent stimulator implantation tends to be informative and advantageous on some occasions. Finally, the SVR model trained by the above features demonstrated good accuracy in outcome prediction, providing us with a more valid assessment tool for patient selection.

## Data availability statement

The data that support the findings of this study are available from the corresponding author upon reasonable request.

## Funding

This research did not receive any specific grant from funding agencies in the public, commercial, or not-for-profit sectors.

## CRediT authorship contribution statement

**Yunhao Wu:** Writing – original draft, Formal analysis, Data curation, Conceptualization. **Yan Li:** Formal analysis, Data curation. **Hongxia Li:** Methodology, Data curation, Conceptualization. **Tao Wang:** Investigation, Data curation. **Peng Huang:** Data curation. **Yiwen Wu:** Supervision. **Bomin Sun:** Validation, Supervision. **Yixin Pan:** Validation, Data curation. **Dianyou Li:** Writing – review & editing, Validation.

## Declaration of competing interest

The authors declare that they have no known competing financial interests or personal relationships that could have appeared to influence the work reported in this paper.
