# Supplementary material for: Prediction of subthalamic stimulation efficacy on isolated dystonia via support vector regression
Source: Heliyon. 2024 May 17;10(10):e31475. doi: 10.1016/j.heliyon.2024.e31475 (PMC11137530; doi:10.1016/j.heliyon.2024.e31475)
Supplement: Multimedia component 1 [file mmc1.docx]

**Supplementary table 1**

1. **Demographic and clinical characteristics of enrolled patients.**

| **Subject No.** | **Gender** | **Age** | **Onset age** | **Duration** | **Baseline BFMDRS** | **BFMDRS motor improvement% (one week)** | **BFMDRS overall improvement% (≥ one year)** |
| --- | --- | --- | --- | --- | --- | --- | --- |
| **1** | M | 29 | 19 | 10 | 68.5 | 50.5 | 70.8 |
| **2** | F | 49 | 47 | 2 | 29 | 5.0 | 6.9 |
| **3** | F | 62 | 59 | 3 | 26 | 33.3 | 46.2 |
| **4** | F | 25 | 23 | 2 | 23 | 94.4 | 91.3 |
| **5** | M | 60 | 56 | 3.5 | 13 | 70.0 | 84.6 |
| **6** | M | 58 | 50 | 8 | 42.5 | 0.0 | 0.0 |
| **7** | F | 59 | 57 | 1.5 | 12 | 85.0 | 66.7 |
| **8** | M | 58 | 55 | 2.5 | 15 | 68.2 | 46.7 |
| **9** | F | 32 | 30 | 2 | 13 | 90.0 | 80.8 |
| **10** | F | 50 | 13 | 37 | 73 | 28.6 | 45.2 |
| **11** | M | 34 | 33 | 1 | 5 | 85.7 | 90.0 |
| **12** | M | 55 | 45 | 10 | 19 | 28.6 | 52.6 |
| **13** | M | 28 | 27 | 1 | 10 | 12.5 | 20.0 |
| **14** | M | 15 | 13 | 1.5 | 32 | 66.7 | 87.5 |
| **15** | M | 66 | 49 | 17 | 17 | 33.3 | 41.2 |
| **16** | F | 39 | 38 | 1 | 33 | 75.0 | 75.8 |
| **17** | F | 53 | 53 | 0.1 | 17 | 35.7 | 52.9 |
| **18** | F | 64 | 63 | 1 | 25 | 72.2 | 80.0 |
| **19** | F | 23 | 12 | 11 | 51 | 75.6 | 72.6 |
| **20** | F | 65 | 59 | 6 | 29 | 47.4 | 27.6 |
| **21** | F | 52 | 47 | 5 | 24 | 0.0 | 20.8 |
| **22** | M | 39 | 36 | 3 | 25.5 | 8.1 | 21.6 |
| **23** | M | 72 | 67 | 5 | 54 | 52.4 | 72.2 |
| **24** | M | 34 | 33 | 1 | 9.5 | 5.9 | 57.9 |
| **25** | F | 67 | 59 | 7.5 | 13 | 70.0 | 46.2 |
| **26** | M | 68 | 62 | 6 | 45 | 58.8 | 71.1 |
| **27** | F | 28 | 27 | 0.5 | 24 | 21.4 | 41.7 |
| **28** | M | 22 | 16 | 6 | 32 | 47.8 | 56.3 |
| **29** | F | 65 | 55 | 10 | 15 | 66.7 | 73.3 |
| **30** | M | 16 | 13 | 3 | 13 | 20.0 | 30.8 |
| **31** | F | 14 | 12 | 2 | 8 | 50.0 | 75.0 |
| **32** | M | 18 | 17 | 1 | 14 | 50.0 | 78.6 |

1. **Demographic and clinical characteristics of patients in test cohort for VTA-based outcome map.**

| **Subject No.** | **Gender** | **Age** | **Onset age** | **Duration** | **Baseline BFMDRS** | **BFMDRS overall improvement% (≥ one year)** |
| --- | --- | --- | --- | --- | --- | --- |
| **1** | M | 38 | 32 | 6 | 91 | 85.2 |
| **2** | M | 46 | 43 | 3 | 18 | 75.0 |
| **3** | M | 33 | 32 | 1 | 7.5 | 80.0 |
| **4** | F | 51 | 49 | 2 | 19 | 60.5 |
| **5** | M | 53 | 46 | 7 | 27 | 66.7 |
| **6** | M | 42 | 32 | 10 | 64 | 85.9 |
| **7** | F | 62 | 58 | 4 | 54 | 88.9 |
| **8** | M | 67 | 59 | 8 | 19 | 44.7 |
| **9** | F | 45 | 40 | 5 | 80 | 90.6 |
| **10** | M | 35 | 29 | 6 | 48 | 75.0 |

**Supplementary table 2**

**Summary of demographic and clinical characteristics.**

|  | **Sex (M/F)** | **Age**  **Md, (P25, P75)** | **Age of onset**  **Md, (P25, P75)** | **Duration (y)**  **Md, (P25, P75)** | **Follow-up time (y)**  **Md, (P25, P75)** |
| --- | --- | --- | --- | --- | --- |
| **Isolated dystonia**  **(n=32)** | 16/16 | 49.50* (28.00, 61.50) | 41.50* (20.00, 55.75) | 3.00* (1.13, 7.13) | 1.04*(1.00, 1.50) |
|  | **Classification** | | | | |
|  | **Symptom forms** | | **Body distributions** | **Long-term clinical outcome (BFMDRS improvement %)** | |
|  | Predominant phasic dystonia: 21  Predominant tonic dystonia: 11 | | Segmental (eyelid, face, neck):19  Generalized (trunk, limbs):13 | Superior outcome (70-100%):14  Moderate outcome (25-69.9%):13  Inferior outcome (0-24.9%):5 | |

Data are presented as Median (25th percentile, 75th percentile)

* Shapiro-Wilk test p<0.05

M/F, Male/Female BFMDRS, Burke–Fahn–Marsden Dystonia Rating scale

**Supplementary table 3**

**Optimal subthalamic stimulation parameters in patients.**

| Subject No. | **Right** | | | | **Left** | | | |
| --- | --- | --- | --- | --- | --- | --- | --- | --- |
|  | Contact | Voltage(V) | Width(ms) | Frequency (Hz) | Contact | Voltage(V) | Width(ms) | Frequency (Hz) |
| 1 | C+2-3- | 3.5 | 60 | 130 | C+9-10- | 4.9 | 80 | 180 |
| 2 | C+1- | 3 | 60 | 130 | C+9- | 3 | 60 | 130 |
| 3 | C+2-3- | 3.55 | 90 | 145 | C+10-11- | 3.75 | 90 | 145 |
| 4 | C+2-3- | 3.15 | 50 | 160 | C+10-11- | 3 | 50 | 160 |
| 5 | C+2-3- | 2.6 | 60 | 160 | C+10-11- | 2.15 | 60 | 130 |
| 6 | C+1-3- | 2.3 | 40 | 120 | C+9-11- | 2 | 40 | 110 |
| 7 | C+2-3- | 2 | 60 | 160 | C+10-11- | 2 | 60 | 160 |
| 8 | C+2-3- | 2.9 | 60 | 160 | C+10-11- | 3.3 | 80 | 160 |
| 9 | C+2- | 3.5 | 90 | 145 | C+10-11- | 3.6 | 70 | 145 |
| 10 | C+1-2- | 3.9 | 60 | 140 | C+10-11- | 3.6 | 60 | 145 |
| 11 | C+2- | 3.45 | 80 | 160 | C+9-10- | 3.15 | 70 | 160 |
| 12 | C+2-3- | 3 | 60 | 135 | C+9-10- | 3 | 60 | 135 |
| 13 | 2-3+ | 3 | 90 | 135 | 10+11- | 3.1 | 90 | 145 |
| 14 | C+2-3- | 3 | 60 | 160 | C+10-11- | 2.55 | 60 | 160 |
| 15 | C+2- | 1.2 | 60 | 135 | C+10- | 1.2 | 60 | 135 |
| 16 | C+2-3- | 3.7 | 60 | 135 | C+10-11- | 3.55 | 60 | 135 |
| 17 | C+2-3- | 2.6 | 60 | 160 | C+10-11- | 2.8 | 60 | 160 |
| 18 | C+2-3- | 3 | 80 | 160 | C+10-11- | 3 | 90 | 160 |
| 19 | 1+2-3- | 3.75 | 100 | 135 | 10+11- | 3.35 | 90 | 145 |
| 20 | C+2-3- | 3 | 60 | 160 | 9+10-11- | 3.5 | 90 | 160 |
| 21 | C+2-3- | 3.25 | 60 | 145 | C+10-11- | 3 | 60 | 145 |
| 22 | C+1-2- | 3.55 | 70 | 145 | C+9-10- | 2.75 | 50 | 145 |
| 23 | 1+2-3- | 2 | 90 | 130 | 9+10-11- | 2 | 90 | 130 |
| 24 | C+2-3- | 2.1 | 60 | 130 | C+10-11- | 2.3 | 60 | 130 |
| 25 | C+2-3- | 3.1 | 90 | 115 | C+10-11- | 3.2 | 90 | 115 |
| 26 | C+2-3- | 2.7 | 60 | 115 | C+10-11- | 2.7 | 60 | 115 |
| 27 | C+2- | 2.75 | 60 | 160 | C+10- | 2.75 | 50 | 160 |
| 28 | 0-1+ | 2.1 | 40 | 150 | 8-9+ | 2.25 | 30 | 150 |
| 29 | C+2-3- | 3.7 | 80 | 145 | C+9-10- | 3.7 | 80 | 145 |
| 30 | C+1-2- | 2.5 | 60 | 130 | C+9-10- | 2.5 | 60 | 130 |
| 31 | 2+3- | 2.75 | 90 | 115 | 10+11- | 3 | 60 | 115 |
| 32 | C+2- | 3.15 | 50 | 125 | C+10-11- | 3.45 | 50 | 125 |
